# Comparison of the Characteristics of Asymptomatic and Presymptomatic Patients with Coronavirus Disease 2019 in the Republic of Korea

**DOI:** 10.1007/s44197-021-00011-7

**Published:** 2021-11-10

**Authors:** Miri Hyun, Ji Yeon Lee, Jae Seok Park, Jin Young Kim, Hyun Ah Kim

**Affiliations:** 1 Division of Infectious Diseases, Keimyung University Dongsan Hospital, Keimyung University School of Medicine, 1035 Dalgubeoldaero, Dalseo-gu, Daegu, 42601 South Korea; 2 Division of Pulmonology, Keimyung University Dongsan Hospital, Keimyung University School of Medicine, 1035 Dalgubeoldaero, Dalseo-gu, Daegu, 42601 South Korea; 3 Department of Radiology, Keimyung University Dongsan Hospital, Keimyung University School of Medicine, 1035 Dalgubeoldaero, Dalseo-gu, Daegu, 42601 South Korea

**Keywords:** Asymptomatic, Chest radiograph, Coronavirus disease 2019, C-reactive protein, Pneumonia, Severe acute respiratory syndrome coronavirus 2

## Abstract

**Purpose:**

This retrospective study aimed to evaluate the baseline characteristics of asymptomatic patients with coronavirus disease 2019 at admission and to follow-up their clinical manifestations and radiological findings during hospitalization.

**Methods:**

Patients with coronavirus disease 2019 who were asymptomatic at admission were divided into two groups—those with no symptoms until discharge (group A) and those who developed symptoms after admission (group B). Patients who could not express their own symptoms were excluded.

**Results:**

Overall, 127 patients were enrolled in the study, of whom 19 and 108 were assigned to groups A and B, respectively. The mean age and median C-reactive protein level were higher in group B than in group A. All patients in group A and one-third of patients in group B had normal initial chest radiographs; 15.8% and 48.1% of patients in groups A and B, respectively, had pneumonia during hospitalization. One patient in group B, whose condition was not severe at the time of admission, deteriorated due to aggravated pneumonia and was transferred to a tertiary hospital.

**Conclusion:**

We summarize the clinical characteristics during hospitalization of patients with coronavirus disease 2019 who were purely asymptomatic at the time of admission. The majority of asymptomatic patients with coronavirus disease 2019 were discharged without significant events during hospitalization. However, it may be difficult to predict subsequent events from initial chest radiographs or oxygen saturation at admission.

## Introduction

In December 2019, there was an outbreak of pneumonia of unknown cause in Wuhan, China. The causative agent was later identified as severe acute respiratory syndrome coronavirus 2 (SARS-CoV-2), and the condition was termed coronavirus disease 2019 (COVID-19) [1]. COVID-19 can cause severe pneumonia that can lead to death in older patients [2]. In contrast, some cases of COVID-19 were asymptomatic. Also, there are cases where there are no symptoms at the time of admission, but symptoms develop during hospitalization [3, 4]. When symptoms do occur, they may not be severe. The symptoms of COVID-19 can be diverse, and among them, there have been studies on asymptomatic patients [3, 5, 6]. The proportion of asymptomatic patients among the total number of patients varied in different reports [5, 7, 8]. Although symptoms may develop during hospitalization, most studies have focused on COVID-19 patients who were asymptomatic at the time of admission [9].

Moreover, most reports on asymptomatic patients included a small number of patients [4, 10]. Long et al*.* [10] reported the clinical and immunological characteristics of 37 asymptomatic patients with COVID-19 for 14 days before a confirmatory test and during hospitalization. Some reports focused on radiologic findings in asymptomatic COVID-19 patients. In Iran, there was a report comparing chest computed tomography (CT) findings in 23 COVID-19 patients who were asymptomatic at the time of admission, and about 40% showed pneumonia [6]. Additionally, although it was a report of asymptomatic patients, among them, patients in long-term care facilities (LTCFs) with difficulty expressing their symptoms were included in the study [11–13]. If patients who have difficulty expressing symptoms are included in the study, it could be estimated that there are more asymptomatic patients than they actually are. Therefore, it is necessary to evaluate the clinical characteristics of patients who were asymptomatic at the time of admission but developed symptoms during hospitalization and those who were continuously asymptomatic. To accurately determine whether symptoms occur, patients in LTCFs should be excluded.

In this study, we aimed to compare the clinical characteristics of asymptomatic patients with COVID-19 at admission who developed symptoms after hospitalization with those who were asymptomatic until discharge from the hospital.

## Materials and Methods

### Ethics Approval

The study design was approved by the Institutional Review Board of Dongsan Medical Center (approval number: 2020-03-027). The requirement for written informed consent was waived owing to the retrospective nature of the study.

### Study Design

We conducted a retrospective observational study of asymptomatic patients with COVID-19 at the time of admission. Patients were admitted to Keimyung University Daegu Dongsan Hospital (KUDDH), South Korea, between February 21 and May 29, 2020. KUDDH is a secondary hospital with 200 beds, temporarily expanded to 450 beds during the COVID-19 outbreak. In the Republic of Korea, SARS-CoV-2 screening tests have been extensively conducted to prevent community transmission of COVID-19. In cases where a person was in contact with a COVID-19-positive individual or had traveled to a country affected by COVID-19, a polymerase chain reaction (PCR) screening test for SARS-CoV-2 was performed regardless of their symptoms [5, 14]. Patients with a history of COVID-19 were also tested for SARS-CoV-2 when they developed symptoms of suspected COVID-19 or were admitted to hospital. If the PCR test for SARS-CoV-2 was positive, patients, including asymptomatic patients, were isolated in hospital or a residential treatment center (RTC). We excluded hospitalized patients when they re-tested positive for SARS-CoV-2 by PCR and those who were symptomatic at the time of admission. Further, we excluded patients < 18 years of age, those in LTCFs, and those transferred to KUDDH after hospitalization in another hospital or RTC. A questionnaire was prepared to determine whether symptoms were present at the time of admission. Every day, doctors interviewed each patient about new symptom development by telephone or during ward rounds. Fever, chills, cough, sputum, rhinorrhea, sore throat, myalgia, headache, diarrhea, dyspnea, and chest pain were included in the questionnaire. Fever was defined as having a body temperature > 37.8 °C. We included patients without symptoms during hospitalization as asymptomatic patients, in group A, and those who developed symptoms during hospitalization as pre-symptomatic patients, in group B.

### Data Collection

We collected data on patient demographics, including sex, age, COVID-19 exposure history, and overseas visits. Initial vital signs, including body temperature, respiratory rate, pulse rate, and systolic blood pressure, were measured. Clinical data, including underlying diseases, symptoms, oxygen demand, laboratory tests, chest radiographs, chest CT findings, and PCR results, were collected. Pneumonia was determined when pneumonic infiltration was observed on chest radiography/CT. The severity of pneumonia was evaluated based on hypoxemia and oxygen demand according to the World Health Organization criteria [15]. Severe pneumonia was defined as one of the following: respiratory rate > 30 breaths/min, severe respiratory distress, or oxygen saturation < 90% on room air.

### Statistical Analyses

Categorical variables were described using frequencies and percentages, while continuous variables were described using means, medians, and interquartile ranges. All statistical analyses were performed using the Statistical Package for the Social Sciences software version 20.0 (SPSS Inc., IBM Corp., Armonk, NY, USA). The Pearson *χ*^2^ test and Fisher’s exact test were used to compare qualitative variables. For continuous variables, normal distribution was tested using the Kolmogorov–Smirnov test. The Mann–Whitney *U* test was performed for data that followed non-normal distributions, and the independent *t* test was performed for data that followed normal distributions. For unadjusted comparisons, *P* < 0.05 was considered statistically significant.

## Results

### Patients’ Baseline Characteristics

**Fig. 1:**
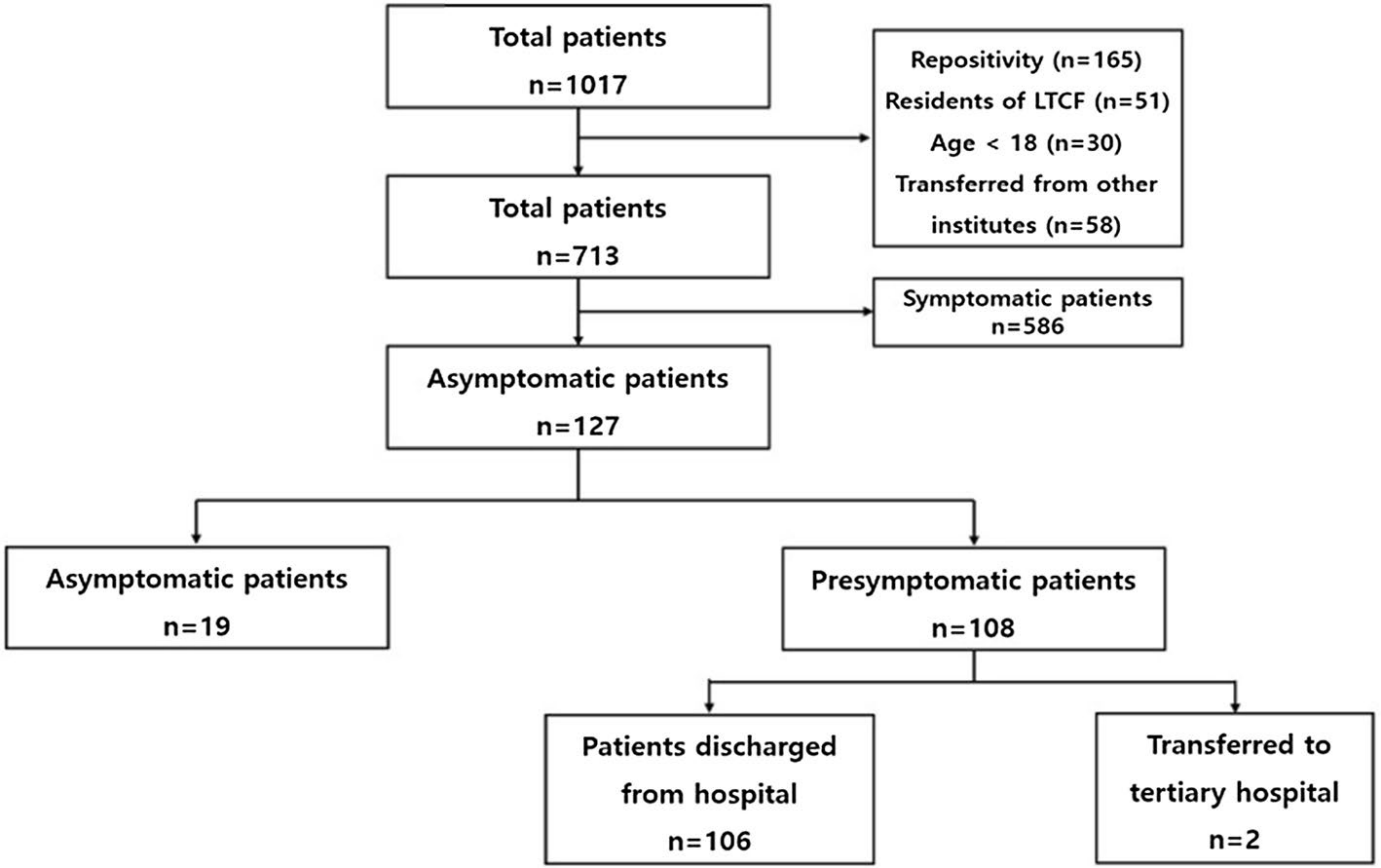
Flowchart of patient enrollment in the study

A total of 1,017 patients were admitted to KUDDH during the study period, of whom 165 patients re-tested positive for SARS-CoV-2. Fifty-one patients who were residents of an LTCF, 30 who were < 18 years of age, 586 who were symptomatic at the time of admission, and 58 who were transferred from other hospitals or RTCs were excluded from the study. The remaining 127 patients were finally included. Of these, 19 patients (group A) had no symptoms during hospitalization, while 108 (group B) developed symptoms (Fig. 1). The demographic characteristics of patients in both study groups are provided in Table 1. The sex distribution was not significantly different between the two groups. Patients in group B had a significantly higher mean age than those in group A. The proportion of patients aged > 65 years was 15.8% in group A and 47.2% in group B (*p* = 0.011). There were no significant differences in underlying diseases between the two groups. However, hypertension, diabetes mellitus, neurological disorders, and malignancies were more common in group B. When screening tests were performed because patients had traveled abroad, more asymptomatic patients were consistently observed and statistically significant (*p* = 0.042).

**Table 1:** Patients’ baseline characteristics

Characteristics	Group A (*n* = 19) *n* (%)	Group B (*n* = 108) *n* (%)	*P* value
Epidemiology			
Male sex	8 (42.1%)	37 (34.3%)	0.34
Age (mean), years	44.32 ± 20.43	59.82 ± 19.52	0.002
Trip abroad	4 (21.1%)	6 (5.6%)	0.042*
Close contact	12 (63.2%)	60 (55.6%)	0.537
Underlying diseases			
Hypertension	2 (15.4%)	37 (35.9%)	0.214*
Diabetes mellitus	1 (7.7%)	16 (15.5%)	0.688*
Hyperlipidemia	1 (7.7%)	9 (8.7%)	0.999*
Chronic lung disease	0 (0.0%)	6 (5.8%)	0.999*
Chronic heart disease	3 (23.1%)	7 (6.8%)	0.083*
Neurological disease	0 (0.0%)	9 (8.7%)	0.595*
Chronic liver disease	0 (0.0%)	1 (1.0%)	0.999*
Autoimmune disease	0 (0.0%)	1 (1.0%)	0.999*
Malignancy	0 (0.0%)	9 (8.7%)	0.595*
Psychiatric disease	0 (0.0%)	2 (1.9%)	0.999*
Pregnancy	0 (0.0%)	1 (1.0%)	0.999*

Group A, patients without symptoms during hospitalization; Group B, patients who developed symptoms

Age is given as the mean ± standard deviation

*Fisher’s exact test

### Patients’ Clinical Characteristics at Admission

**Fig. 2:**
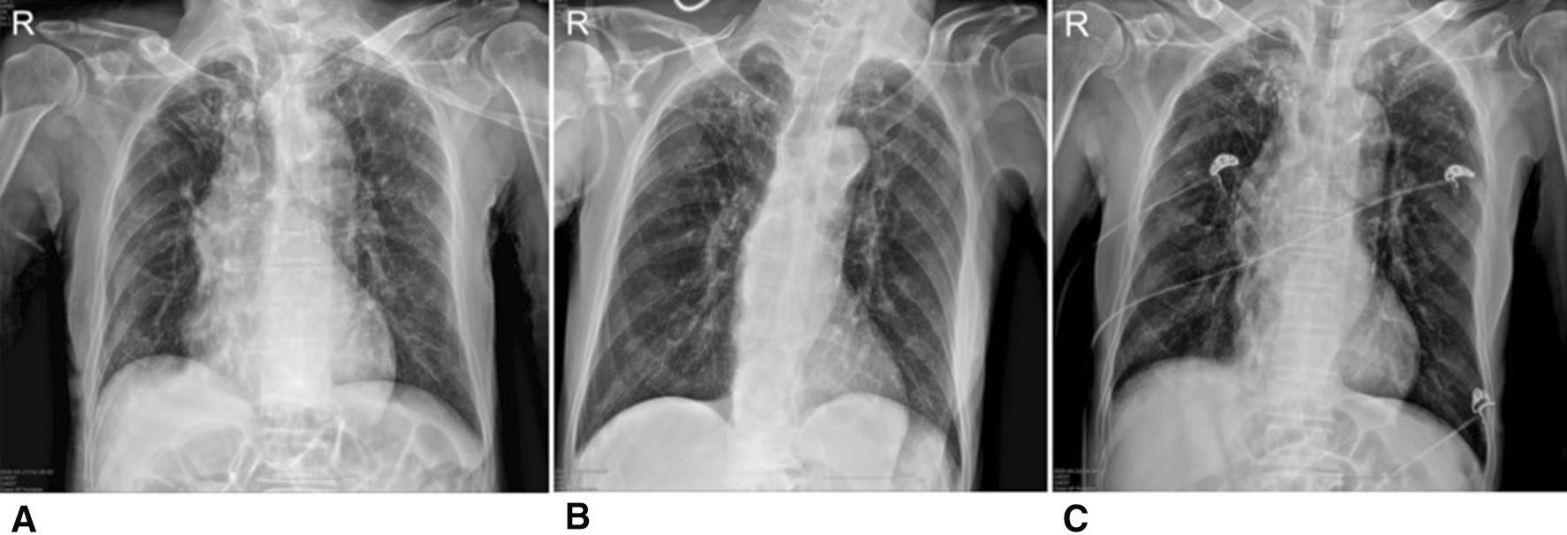
Serial chest radiographies of patient C. **A** Shows post-inflammatory fibrosis and nodules, at both upper lobes, GGO at both lower lung field (Esp. LLL) at initial chest radiograph. **B** Taken 5 days after shows no interval change of pneumonia at both lower lungs. **C** Taken 3 weeks after shows slightly improvement of pneumonia

**Fig. 3:**
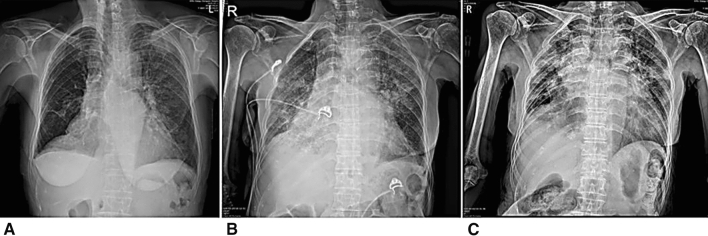
Serial chest radiographies of patient D. **A** Shows mass like consolidation at LUL, diffuse GGO at left lung, suggestive of COVID pneumonia at initial chest radiograph. **B** Taken 3 days after shows aggravation of pneumonia, or increased extent of pneumonia, diffuse GGO and consolidation at both lung fields. **C** Taken 10 days after shows slightly more aggravated pneumonia at both lung field

Initial vital signs were not significantly different between the two groups. The laboratory findings of patients in both study groups are summarized in Table 2. The median initial absolute lymphocyte count was lower in group B, without significance. The median initial C-reactive protein (CRP) and lactate dehydrogenase levels were higher in group B with significance. All patients in group A had normal initial chest radiographs, while 32.4% of patients in group B had pneumonia on initial chest radiographs. Initial chest CT scans were performed in 13 patients in group A and 69 patients in group B. In two patients in group A (15.4%, 2/13) and 26 patients in group B (37.7%, 26/69), pneumonia was detected on initial chest CT scans. Among 26 patients in group B whose CT findings were pneumonia, 14 had pneumonic infiltration on initial chest X-rays and 12 had normal findings. An 84-year-old man (patient C) in group B was categorized as having severe pneumonia. At the time of admission, the patient’s initial oxygen saturation was 86%. Pneumonia was detected on the initial chest radiograph (Fig. 2). Chest CT was not performed. The patient visited the emergency room due to altered mental status; brain CT was performed, and the patient was diagnosed with cerebral infarction in the right middle cerebral artery territory. The patient was diagnosed with COVID-19 by a surveillance test conducted before admission. An 80-year-old woman (patient D) in group B, who underwent lobectomy for lung cancer, was categorized as having non-severe pneumonia at the time of admission and had pneumonia on the initial chest radiograph (Fig. 3). The patient’s initial oxygen saturation was 95%.

**Table 2:** Initial laboratory and radiological findings

Findings	Group A (*n* = 19) *n* (%)	Group B (*n* = 108) *n* (%)	*P* value
Laboratory findings			
WBC (µL)	5533.68 ± 1316.13	5999.87 ± 1844.81	0.294
Hemoglobin (g/dL)	13.19 ± 2.13	12.63 ± 1.66	0.194
Platelet (µL)	242.37 ± 67.29	247.58 ± 83.32	0.797
ANC	3,189.11 ± 975.87	3,795.60 ± 1,555.01	0.104
ALC	1,744.58 ± 459.59	1,661.75 ± 649.42	0.596
PT (INR)	1.0 (0.96–1.03)	0.97 (0.92–1.03)	0.103
PT (sec)	11.70 (11.30–12.20)	11.65 (11.00–12.20)	0.429
aPTT (sec)	29.30 (27.30–31.70)	26.90 (25.33–28.75)	0.001
BUN (mg/dL)	12.00 (11.00–15.00)	14 (11–18)	0.157
Creatinine (mg/dL)	0.72 (0.62–0.92)	0.75 (0.63–0.89)	0.625
Albumin (g/dL)	4.40 (4.40–4.60)	4.20 (3.80–4.40)	0.001
AST (U/L)	18 (17–24)	20.50 (16–27.25)	0.568
ALT (U/L)	14 (12–20)	19 (13–27.25)	0.08
CRP (mg/dL)	0.03 (0.01–0.11)	0.10 (0.03–0.50)	0.011
LDH (mg/dL)	358 (301.50–400.00)	412 (354–483.50)	0.016
Radiological findings			
Abnormal initial chest radiograph	0 (0.0%)	35 (32.4%)	0.004
Abnormal initial chest CT scan	2 (15.4%)	26 (37.7%)	0.201*

Group A, patients without symptoms during hospitalization; Group B, patients who developed symptoms

*ALC* absolute lymphocyte count; *ALT* alanine aminotransaminase; *ANC* absolute neutrophil count; *aPTT* activated partial thromboplastin time; *AST* aspartate aminotransferase; *BUN* blood urea nitrogen; *CRP* C-reactive protein; *CT* computed tomography; *LDH* lactate dehydrogenase; *PT* prothrombin time; *WBC* white blood cell

*Fisher’s exact test

### Follow-Ups During Hospitalization

**Fig. 4:**
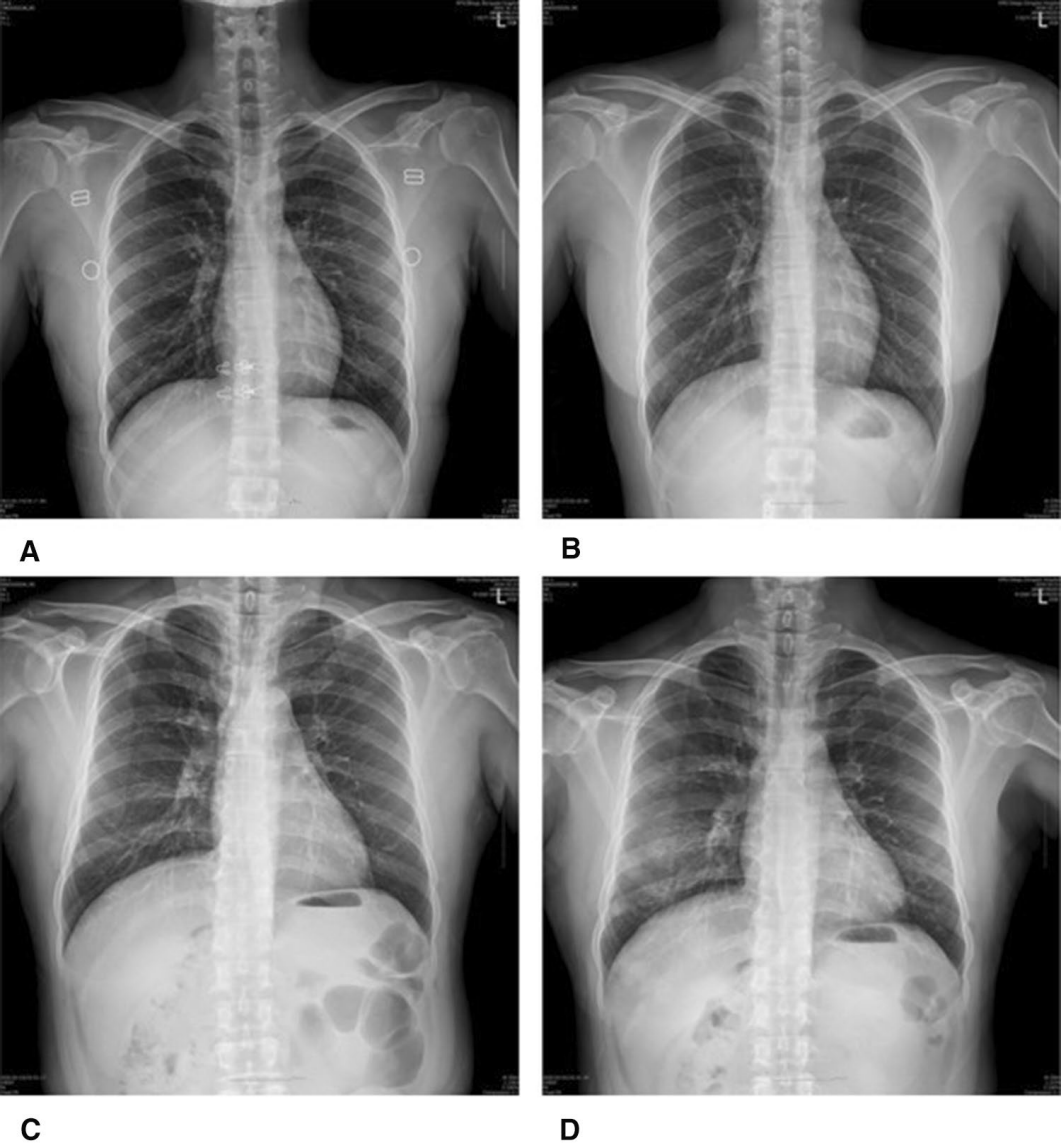
Serial chest radiographic findings of COVID-19 patients. **A** A 28-year-old woman without any symptoms. Normal finding of chest radiograph. **B** Same patient as in image (**A**), taken 4 days after, shows normal finding of chest radiograph. **C** A 59-year-old man with symptoms of COVID-19 during hospitalization. Normal finding of chest radiograph. **D** Same patient as in image (**C**), taken 7 days after, shows newly developed consolidation and GGO at both lower and right mid-lung field

The common symptoms that patients in group B developed during hospitalization are listed in Table 3. Cough was the most common symptom (65.4%), followed by sputum (47.1%), fever (43.3%), myalgia (42.3%), and sore throat (34.3%). The median duration of hospital stay was 13 and 17.5 days in groups A and B, respectively. Follow-up chest radiographs were performed in 18 patients in group A and 102 patients in group B. Two patients in group A had new pneumonia findings. Three patients with pneumonic infiltration and eight patients with normal findings on initial chest radiographs in group B had aggravated radiological findings on follow-up chest radiographs. Follow-up chest CT were performed in 2 patients in group A and 18 patients in group B. One patient in group A and 2 patients in group B had new pneumonic infiltrations in chest CT. During hospitalization, three patients (15.8%) in group A and 52 patients (48.1%) in group B had pneumonia on radiological tests, including chest radiography/CT (Tables 4 and 5). In group B, two patients were transferred to a tertiary hospital (2/127, 1.57%). Patient D’s condition deteriorated due to aggravated pneumonia (Fig. 3). A high-flow nasal cannula was used during hospitalization and the patient was transferred to a tertiary hospital (Fig. 3). One patient was transferred to determine the etiology of pericardial effusion. Patient C showed improvement of pneumonia on a follow-up chest radiograph (Fig. 2). We presented chest X-ray images of two other patients who were followed up during hospitalization (Fig. 4).

**Table 3:** Symptoms that patients developed in Group B

Symptoms	Total (*n* = 108), *n* (%)
Cough	68 (65.4%)
Sputum	49 (47.1%)
Fever	45 (43.3%)
Myalgia	44 (42.3%)
Sore throat	36 (34.3%)
Headache	35 (33.7%)
Chills	35 (33.7%)
Diarrhea	32 (30.8%)
Rhinorrhea	24 (23.1%)
Dyspnea	24 (23.1%)
Chest pain	9 (8.7%)

Group B, patients who developed symptoms during hospitalization

**Table 4:** Chest radiographic findings in asymptomatic and pre-symptomatic COVID-19 patients

Findings	Group A (*n* = 19) *N*	Group B (*n* = 108) *n*
Pneumonia on initial chest X-ray	0	35
Patients of performed follow-up chest X-ray	18	102
Newly developed pneumonia on follow-up chest X-ray	2	8
Aggravated pneumonia on follow-up chest X-ray	0	3

COVID-19, coronavirus disease 2019. Group A, patients without symptoms during hospitalization; Group B, patients who developed symptoms

*CT* computed tomography

**Table 5:** Chest CT findings in asymptomatic and pre-symptomatic COVID-19 patients

Findings	Group A (*n* = 13) *n*	Group B (*n* = 69) *n*
Pneumonia on initial chest CT	2	26
Patients of performed follow-up chest CT	2	18
Newly developed pneumonia on follow-up chest CT	1	2
Aggravated pneumonia on follow-up chest CT	1	1

COVID-19, coronavirus disease 2019. Group A, patients without symptoms during hospitalization; Group B, patients who developed symptoms

*CT* computed tomography

## Discussion

We compared the clinical characteristics between pre-symptomatic and asymptomatic patients with COVID-19. The pre-symptomatic group had more underlying diseases. The mean age and initial CRP and lactate dehydrogenase levels were higher in pre-symptomatic patients. The mean absolute lymphocyte count and median initial albumin level were lower in pre-symptomatic patients. Initial chest radiographs were normal in asymptomatic patients. Pneumonia findings on initial and follow-up chest radiographs were observed in approximately 50% of pre-symptomatic patients. Considering the cases of patients C and D, even on hospital admission of asymptomatic patients with COVID-19, the evaluation of patients’ prognosis based on criteria, such as abnormal chest radiographs and decreased oxygen saturation, may be difficult.

SARS-CoV-2 screening tests were conducted worldwide. In other countries, asymptomatic patients had been identified through SARS-CoV-2 PCR screening tests for individuals who were in close contact with patients with COVID-19. Most of the studies on asymptomatic patients with COVID-19 focused on infectivity and screening importance rather than clinical characteristics [4, 8, 16, 17]. In the Republic of Korea, SARS-CoV-2 screening tests have been extensively conducted among family members, company staff, and the general population to prevent community transmission of COVID-19. In cases where a person was in contact with a COVID-19-positive individual, had traveled to a country affected by COVID-19, or had resided at an LTCF where COVID-19-positive patients were hospitalized, a PCR screening test for SARS-CoV-2 was performed regardless of their symptoms [5, 14]. If the PCR test was positive, patients, including asymptomatic patients, were isolated in hospital or an RTC. This governmental guideline provided us with an opportunity to observe and examine asymptomatic or pre-symptomatic patients during their hospitalization. Unlike RTCs, we regularly checked vital signs, laboratory tests, chest radiographs, and chest CT scans.

The proportion of asymptomatic infections with COVID-19 varied between studies, depending on the country, medical institution, the tested group, patient epidemiology, and intensive surveillance [7, 8, 10, 17, 18]. In a review article on COVID-19, it was reported that the proportion of asymptomatic patients during COVID-19 was about 4%, but in this study, it was about 22% [19]. This is thought to be due to more intensive surveillance in Republic of Korea than in other countries. According to studies published so far, asymptomatic COVID-19 patients were younger and had fewer underlying diseases, and chest CT was more often normal. In addition, patients with pre-symptomatic COVID-19 were more likely to be elderly, have hypertension, have abnormal findings on chest CT, especially with consolidations [19, 20]. We found that pre-symptomatic patients had more underlying diseases.

A study in Saudi Arabia classified patients with symptoms and those without symptoms at COVID-19 diagnosis, and more asymptomatic patients tended to have diabetes mellitus [13]. Based on our study and the results of the Saudi Arabian study, even if patients with diabetes mellitus had no symptoms at the time of diagnosis of COVID-19, the possibility of them developing symptoms later should be considered. Diabetes mellitus is associated with impaired immune responses, including reduced response to T cells, disorders of humoral immunity, and lower secretion of inflammatory cytokines [21, 22]. In case of underlying diseases, such as hypertension or coronary artery disease, patients may require intensive care [23]. The association between hypertension and the severity of COVID-19 has been evaluated in several reports [23, 24]. SARS-CoV-2 binds to angiotensin-converting enzyme 2 (ACE2). SARS-CoV-2 shows its pathogenicity by attacking type II alveolar epithelial cells expressing ACE2. Loss of ACE2 may affect higher angiotensin II and angiotensin-(1–7) tone. Angiotensin II promotes vasoconstriction, oxidative stress, inflammation, and fibrosis [25].

As the COVID-19 pandemic continues, the redistribution of medical resources is necessary, and it is highly likely that interest in asymptomatic patients will decrease. However, even among asymptomatic patients, symptoms develop during hospitalization and progress to severe pneumonia [16]. When the symptoms of pre-symptomatic COVID-19 patients were analyzed, cough was the most common in most cases, depending on the reports [26, 27]. In other study, fever was the most prevalent new symptom in pre-symptomatic COVID-19 patients [16]. In our study, cough was the most common, followed by sputum, and less than half of the patients complained of fever. Developed symptoms might vary depending on the presence of viral pneumonia, immune status, underlying diseases, or patients’ age.

Elevated CRP levels may be associated with an overproduction of inflammatory cytokines, which in turn may lead to tissue destruction. Elevated plasma CRP levels are associated with disease severity in patients with COVID-19, and higher CRP levels correlated with longer durations of hospitalization [23, 28]. There was also a report about the correlation between CRP level and infiltrations in chest CT [20]. In addition, lymphopenia is a risk factor for prolonged hospitalization [29]. In our study, pre-symptomatic patients had a longer hospital stay. This may be related to laboratory findings, but may also be related to the COVID-19 response guidelines of the Korean Centers for Disease Control and Prevention [30]. In the early stages of the response to COVID-19 in the Republic of Korea, a PCR test was performed when the patients’ symptoms had improved, and at least two negative PCR results were required before discharge. Therefore, if the patients who had no symptom at admission, developed symptoms during hospitalization, the timing of the PCR test was inevitably delayed.

Chest radiography is a valid diagnostic tool for COVID-19 [31]. In a Singapore study including only asymptomatic and mild symptomatic COVID-19 patients, only 2% of the patients showed pneumonic infiltration on chest radiographs. Among them, only 0.2% of those patients were needed to supplemental oxygen therapy and they all had symptoms. So this team recommended that screening of chest radiograph is not indicated in asymptomatic COVID-19 patients under 60 years old [32]. However, even in asymptomatic patients, pneumonia is often detected on chest radiographs, and pulmonary sequelae such as pulmonary fibrosis are rare after COVID-19 infection [2]. Therefore, if possible, it is recommended to conduct a chest radiograph at the time of diagnosis and follow-up if pneumonia is present. According to several studies, the diagnosis of pneumonia may differ depending on whether the imaging modality is chest radiography or chest CT [20, 32, 33]. In a Turkish study, 81 patients with traumatic injuries were incidentally diagnosed with COVID-19 pneumonia on initial chest CT scans with no symptoms. Among them, 21% with no symptoms on admission were categorized as severe COVID-19 during hospitalization [3]. In a study including patients < 65 years of age in Wuhan, approximately 50% of asymptomatic COVID-19 cases had pneumonia on chest CT scans [34]. Chest CT has higher sensitivity for COVID-19 than reverse transcriptase-PCR 20. Also, chest CT is recommended in some reports to estimate the extent of pneumonia in patients with suspected COVID-19 pneumonia on chest X-ray [35]. In our study, all asymptomatic patients had normal findings on initial chest radiographs, and two asymptomatic patients (15.38%) had pneumonia on initial chest CT scans. Over 32% and 37.7% of the pre-symptomatic patients had pneumonic infiltration on initial chest radiographs and chest CT, respectively. As pneumonia is detected even if there are no clinical or mild clinical symptoms, it can be seen that COVID-19 mainly causes lower respiratory tract infections, unlike influenza, which mainly causes upper respiratory tract infections [36, 37].

The mortality rate of asymptomatic or pre-symptomatic patients with COVID-19 varied from 4.6 to 33%, for these studies were conducted that included residents of LTCFs [11, 16]. Most LTCF residents could not express their symptoms, which is different from patients who are asymptomatic. LTCF residency is already known to be a risk factor for COVID-19 exacerbation [38]. If a study of asymptomatic patients involves LTCF residents, the results may seem worse than they actually are. Therefore, a high proportion of relatively young and healthy individuals was included in our study. These factors may have influenced our outcomes.

This study had several limitations. First, this was a retrospective observational study, and the symptom status was determined from medical records alone. Hence, causality between the study exposures and outcomes could not be established. Second, subjective symptoms, such as chills, myalgia, and headaches, may have been difficult to detect in older patients. Third, follow-up chest imaging was not performed in all patients. Finally, immunological tests, such as those used to detect COVID-19 antibodies, were not performed. Despite these limitations, this study’s findings contribute significantly to the current literature as they emphasize the necessity of careful monitoring of asymptomatic patients with COVID-19.

## Conclusion

In this study, we identified the characteristics of asymptomatic and pre-symptomatic patients with COVID-19 in the Republic of Korea. Most COVID-19 patients who had no symptom at the time of admission were discharged without significant events during hospitalization. However, considering that one patient with no symptom and no hypoxia at the time of admission was transferred to a tertiary hospital due to worsening pneumonia, it will be needed to monitor the patients with underlying diseases who do not have any symptom and hypoxia at the time of admission. Therefore, it may be difficult to accurately predict subsequent events from initial chest radiographs and oxygen saturation at the time of admission.

## Data Availability

The datasets used and/or analyzed during the current study available from the corresponding author on reasonable request.

## Abbreviations

COVID-19: Coronavirus disease 2019
CRP: C-reactive protein
CT: Computed tomography
KUDDH: Keimyung University Daegu Dongsan Hospital
LTCF: Long-term care facility
PCR: Polymerase chain reaction
RTC: Residential treatment center
SARS-CoV-2: Severe acute respiratory syndrome coronavirus 2
